# Predicting Response to Antibody Drug Conjugates: A Focus on Antigens’ Targetability

**DOI:** 10.1093/oncolo/oyad246

**Published:** 2023-09-04

**Authors:** Liliana Ascione, Edoardo Crimini, Dario Trapani, Antonio Marra, Carmen Criscitiello, Giuseppe Curigliano

**Affiliations:** Division of Early Drug Development, IEO, European Institute of Oncology, IRCCS, Milan, Italy; Department of Oncology and Hematology (DIPO), University of Milan, Milan, Italy; Division of Early Drug Development, IEO, European Institute of Oncology, IRCCS, Milan, Italy; Department of Oncology and Hematology (DIPO), University of Milan, Milan, Italy; Division of Early Drug Development, IEO, European Institute of Oncology, IRCCS, Milan, Italy; Department of Oncology and Hematology (DIPO), University of Milan, Milan, Italy; Division of Early Drug Development, IEO, European Institute of Oncology, IRCCS, Milan, Italy; Division of Early Drug Development, IEO, European Institute of Oncology, IRCCS, Milan, Italy; Department of Oncology and Hematology (DIPO), University of Milan, Milan, Italy; Division of Early Drug Development, IEO, European Institute of Oncology, IRCCS, Milan, Italy; Department of Oncology and Hematology (DIPO), University of Milan, Milan, Italy

**Keywords:** antibody-drug conjugates, predictive biomarkers, molecular imaging, molecular assay

## Abstract

Antibody-drug conjugates (ADCs) represent a cornerstone in the treatment of many cancers nowadays. ADCs fulfill their function by binding a target on tumor cell membrane to deliver a cytotoxic payload; in addition, those moieties capable of crossing cancer cell membranes can achieve near-by cells that do not express the target antigen, exerting the so-called “bystander” cytotoxic effect. The presence of a specific target antigen expressed on cancer cells has been for long considered crucial for ADCs and commonly required for the inclusion of patients in clinical trials with ADCs. To date, only ado-trastuzumab-emtansine, fam-trastuzumab deruxtecan-nxki, and mirvetuximab soravtansine-gynx are approved according to the expression of a target antigen in solid tumors, while the clinical use of other ADCs (eg, sacituzumab govitecan) is not conditioned by the presence of a specific biomarker. Given the ever-growing number of approved ADCs and those under investigation, it is essential to find new biomarkers to guide their use, especially in those settings for which different ADCs are approved to establish the best therapeutic sequence based on robust biomarkers. Hence, this work addresses the role of target antigens in predicting response to ADCs, focusing on examples of antigens’ targetability according to their expression on cancer cells’ surface or to the presence of specific target aberrations (eg, mutation or over-expression). New methods for the assessment and quantification of targets’ expression, like molecular imaging and in vitro assays, might be key tools to improve biomarker analysis and eventually deliver better outcomes by refined patient selection.

**Implications for Practice**

This article provides an overview of biomarkers predicting response to antibody-drug conjugates.

## Introduction

**Figure 1.**
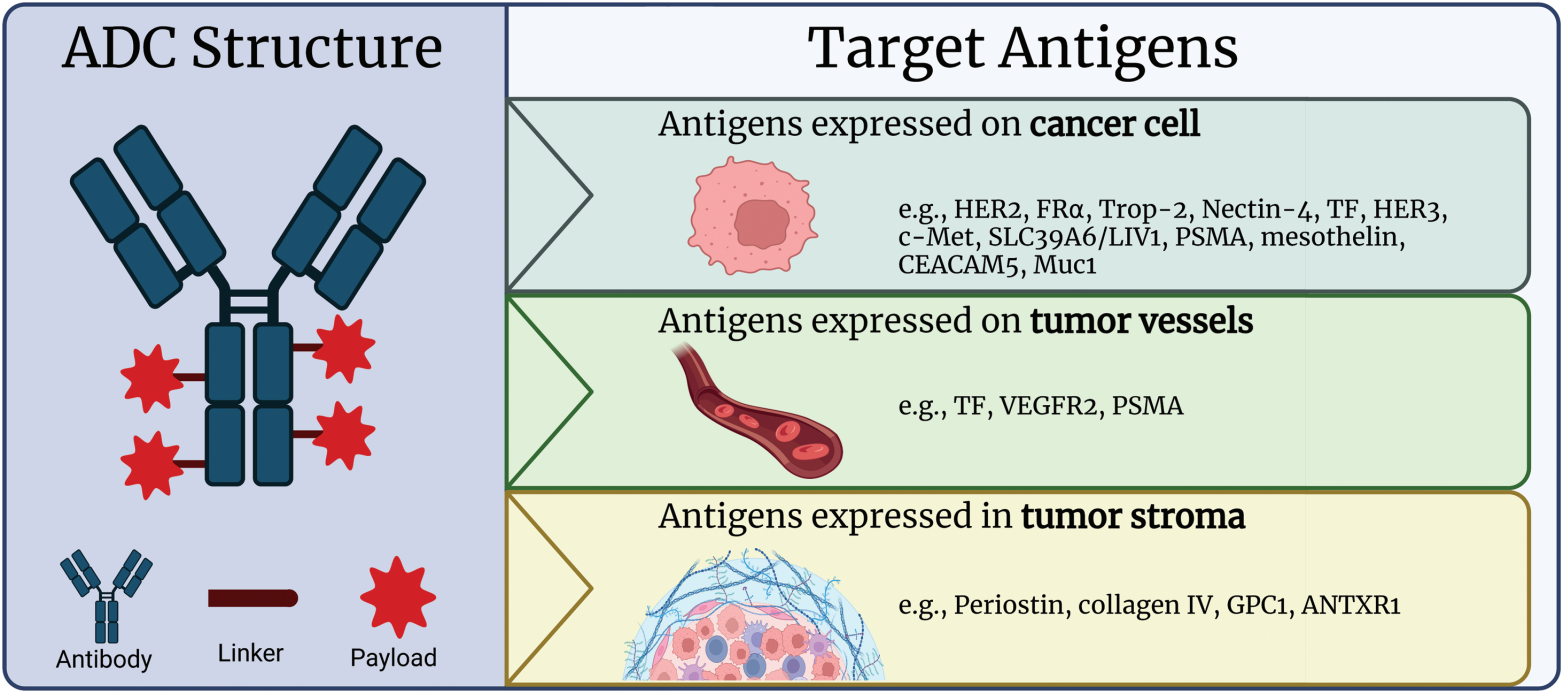
ADC structure and target antigens. (**A**) ADCs are composed of an antibody conjugated to a cytotoxic payload by the mean of a linker. Antibodies are mainly IgG, especially IgG1. Linkers should guarantee high stability in circulation and the release of the cytotoxic moiety in targeted tissues. They are classified into cleavable and non-cleavable: cleavable linkers release the payload after the exposure to specific chemical/enzymatic stimuli (eg, low pH or proteolytic cleavage), while non-cleavable linkers depend on the complete degradation of the ADC in the lysosome, thus being more stable in circulation and causing less off-target toxicity. Most employed chemotherapeutic agents for ADC design are tubulin disrupting agents (eg, auristatin derivatives like MMAE, and maytansinoid derivatives such as DM1) and DNA damaging agents (eg, calicheamicins, duocarmycins, and topoisomerase I inhibitors). (**B**) Most studied target antigens are proteins (over)expressed on cancer cells’ surface. (**C**-**D**) ADCs-targeting antigens found in the tumor microenvironment have also been designed, possibly widening ADCs’ spectrum of activity, since cancer cells rely on tumor stroma and tumor vasculature for their growth and survival. Abbreviations: ADC: antibody drug conjugate; HER2: human epidermal growth factor receptor 2; FRα: folate receptor α; TF: tissue factor; Trop-2: trophoblast cell surface antigen-2; HER3: human epidermal growth factor receptor 3; CEACAM5: carcinoembryonic antigen-related cell adhesion molecule 5; Muc1: mucin 1; SLC39A6: solute carrier family 39 member 6; VEGFR2: vascular endothelial growth factor receptor 2; PSMA: prostate-specific membrane antigen; GPC1: glypican 1; ANTXR1: anthrax toxin receptor 1; MMAE: monomethyl auristatin E. Created with BioRender.com (2023).

**Figure 2.**
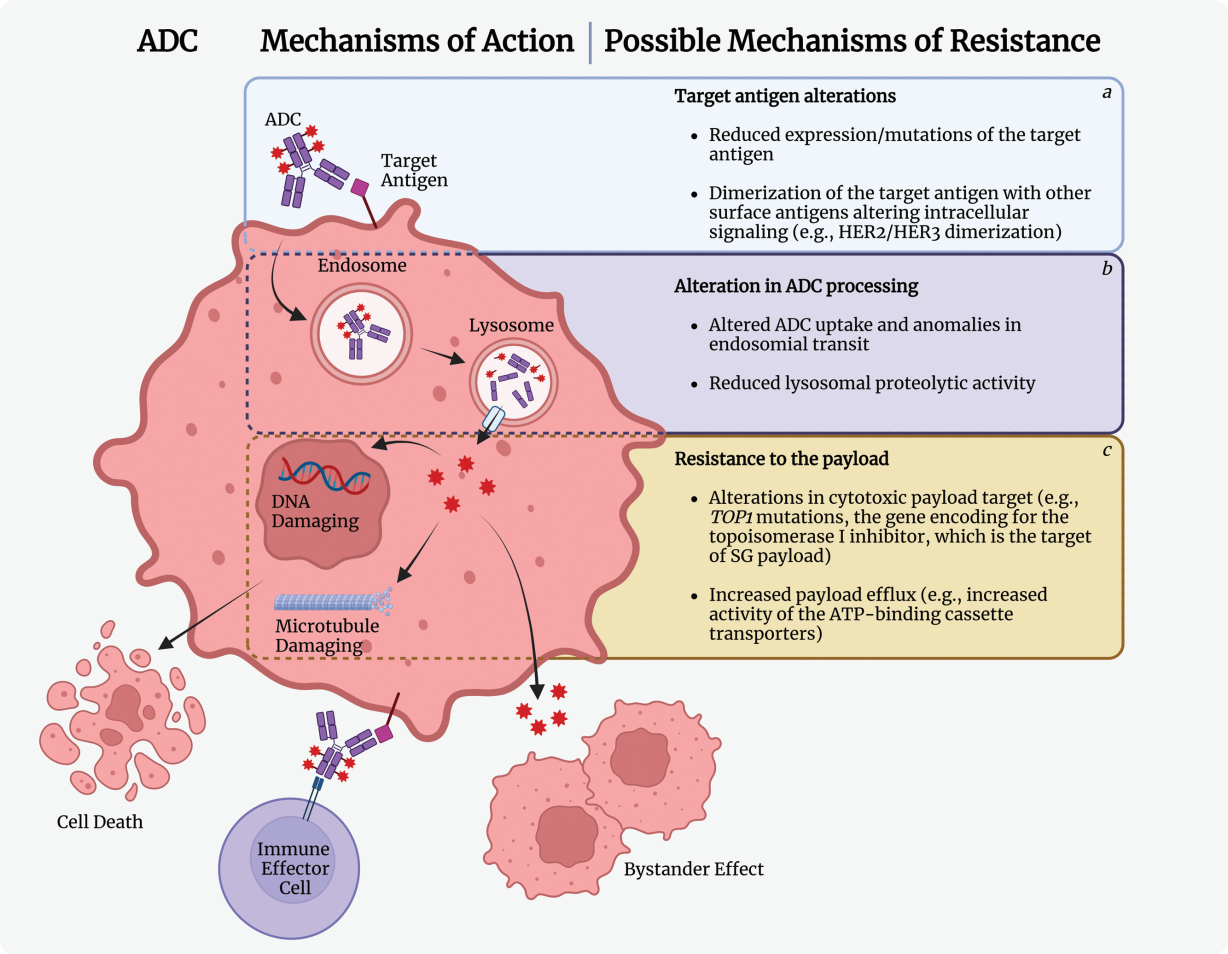
ADC mechanisms of action and putative mechanisms of resistance. Once the ADC binds its target on the cancer cell membrane, the ADC-target complex is internalized via endocytosis to form an endosome. At this early stage, ADCs with cleavable linkers can already release a certain amount of the payload. Then, the endosome fuses with the lysosome where ADC processing is completed through its degradation: the cytotoxic moiety released in the cytoplasm from both cleavable and non-cleavable linkers can exert its activity damaging cellular DNA or disrupting microtubules, depending on the specific chemotherapeutic agent used, resulting in cancer cell death by apoptosis. If the released payload can cross cell membrane, the ADC can enhance its cytotoxic activity exerting the so-called “bystander effect,” since the moiety can reach those cells that do not express the target antigen but localized near-by, as a paracrine chemotherapy delivery. The Ab part of the ADC bound on the cancer cell surface engages the immune system too, activating immune effector cells in diverse ways, namely, the ADCC, the ADCP, and the CDC. In addition, ADCs’ anticancer activity also relies on targeting receptors involved in intracellular signaling (eg, HER2), thus altering key stimuli for tumor growth and survival. Putative mechanisms of resistance to ADCs are described in boxes a, b, and c. Abbreviations: ADC: antibody drug conjugate; HER2: human epidermal growth factor receptor 2; HER3: human epidermal growth factor receptor 3; SG: sacituzumab govitecan; TOP1: DNA topoisomerase I; ATP: adenosine triphosphate; TME: tumor microenvironment; ADCC: antibody-dependent cell cytotoxicity; ADCP: antibody-dependent cellular phagocytosis; CDC: complement-dependent cytotoxicity. Created with BioRender.com (2023).

Antibody-drug conjugates (ADCs) represent a next-generation strategy to deliver chemotherapeutics into cancer cells, namely a sort of “smart chemotherapy.”^1^ ADCs are constituted of 3 components: a monoclonal antibody (mAb) backbone (1) connected to a cytotoxic payload (2) by a linker molecule (3) (Fig. 1). Once the mAb binds its target (ie, commonly the extracellular domain of a transmembrane protein), ADC-antigen complexes are internalized via endocytic pathway, thus delivering the cytotoxic payload(s) to cancer cells. ADCs can deliver a variable quantity of cytotoxins, as described by the drug-to-antibody ratio, that is the average number of payload molecules conjugated to a single mAb.^2^ Due to ADCs’ complex structure, mechanisms of resistance are likewise multifaceted and still not fully undisclosed^3,4^ (Fig. 2).

Selectivity of ADCs has been considered strictly tied to the presence of a target antigen on cancer cells, as delivered through an antigen-specific mAb. Examples of biomarker-based ADCs’ development date back to the 1990s’ when initial clinical trials investigating this class of drug had the expression of the target antigen as a requirement for study enrollment (eg, KS1/4-methotrexate immunoconjugate,^5^ BR96-Doxorubicin^6^). First ADCs for solid tumors were eventually not further developed in clinical trials based on relevant off-target toxicities due to linker instability, which prevented dose-escalation thus limiting efficacy.^7^ Notably, off-target toxicities represent one of the main ADCs’ limitations, among others (Table 1).^8^ Subsequently, Sievers et al evaluated an anti-CD33 immunoconjugate, gemtuzumab ozogamicin, for the treatment of relapsed/refractory CD33+ acute myeloid leukemia in phase I and II trials. In both studies, the expression of the CD33 antigen on the surface of leukemic blast cells was necessary for the enrollment.^9,10^ In May 2000, gemtuzumab ozogamicin became the first ADC granted for approval by the Food and Drug Administration (FDA); however, due to safety issues including early mortality, hematologic toxicity, and hepatotoxicity (eg, severe veno-occlusive syndromes),^11,12^ gemtuzumab ozogamicin was temporarily withdrawn in 2010, and reapproved by FDA in 2017 for the treatment of both newly diagnosed and relapsed/refractory acute myeloid leukemia using a different administration schedule and a lower dose.^13-15^

**Table 1.** Advantages and limitations of antibody-drug conjugates.

Advantages	Targeted delivery of the cytotoxic payload
	Increased therapeutic window
	The presence of linkers should ensure ADC stability in the bloodstream
	Limited safety issues, as the target antigen is selectively expressed by cancer cells with minimal/no expression on “normal cells”
Limitations	The need of specific tests for the target antigen detection to select pts for ADCs is still debated (ie, lack of biomarkers of response)
	Heterogeneity of the target antigen expression
	Off-target toxicity due to premature release of the cytotoxic moiety or to the expression of the target antigen on “normal cells”
	Suboptimal amount of chemotherapeutic agent achieving cancer cells.

Abbreviations: ADCs: antibody-drug conjugates; pts: patients.

To date, only 3 ADCs for solid tumors are approved based on the presence of a specific target: ado-trastuzumab-emtansine (T-DM1), fam-trastuzumab deruxtecan-nxki (T-DXd), and mirvetuximab soravtansine (MIRV; Table 2).

**Table 2.** Efficacy of antibody-drug conjugates approved by FDA for the treatment of solid tumors.

Target	Drugs	Drug characteristics	FDA approval indication	Clinical trial (phase)	Primary efficacy endpoints^a^	Key secondary efficacy endpoints^a^	Target Ag alteration/expression required
HER2	Ado-trastuzumab-emtansine (T-DM1)	Payload: DM1, maytansinoid, microtubule inhibitor Linker: uncleavable DAR = 3.5	HER2+mBC, 2L (progressing to taxane and trastuzumab)	EMILIA (III): randomized vs. capecitabine + lapatinib	PFS 9.6 vs. 6.4 mo (HR 0.65, 95% CI, 0.55-0.77; *P* < .001); OS 29.9 vs. 25.9 mo (HR 0.75; 95% CI, 0.64-0.88; *P* < .001)	ORR 43.6% vs. 30.8% (*P* < .001) DOR 12.6 vs. 6.5 mo	HER2^b^ by IHC 3+ or 2+/ISH+
			HER2 + eBC, adjuvant therapy (if non-pCR after neoadjuvant therapy)	KATHERINE (III): randomized vs. trastuzumab	iDFS 88.3% vs. 77.0% (HR 0.50, 95% CI, 0.39-0.64; *P* < .001)	DRFI 83.0% vs. 89.7% (HR 0.60, 95% CI, 0.45-0.79)	HER2^b^ by IHC 3+ or 2+/ISH+
	Fam-trastuzumab deruxtecan-nxki (T-DXd)	Payload: DXd, campothecin, TOPO1 inhibitor Linker: cleavable DAR = 8	HER2 + mBC, ≥3L	DESTINY-Breast01 (II): 2-part, single group	ORR 61.4%	PFS 19.4 mo (95% CI, 14.1-NE) OS 28.4 mo (95& CI, 23.1-NE)^d^ DOR 20.8 mo (95% CI, 15.0-NE) DCR 97.3% (95% CI, 93.8-99.1) CBR 76.1% (95% CI, 69.3-82.1)	HER2^b^ by IHC 3+ or 2+/ISH+
			HER2 + mBC, 2L (after progressing to trastuzumab and taxane)	DESTINY-Breast03 (III): randomized vs. T-DM1	PFS 28.8 vs. 6.8 mo (HR 0.33, 95% CI, 0.26-0.43; *P* < .0001)^e^	OS NR vs. NR (HR 0.64; 95% CI, 0.47-0.87; *P* = .0037)^e^ ORR 79% vs. 35%^e^	HER2^b^ by IHC 3+ or 2+/ISH+
			HER2-low mBC, after 1 or 2 lines of CHT	DESTINY-Breast04 (III): randomized vs. TPC	PFS (HR + cohort) 10.1 vs. 5.4 mo (HR 0.51, 95% CI, 0.40 to 0.64; *P* < .001)	PFS (overall population): 9.9 vs. 5.1 mo (HR 0.50; 95% CI, 0.40 to 0.63; *P* < .001) OS (HR + cohort): 23.9 vs. 17.5 mo (HR 0.64; 95% CI, 0.48 to 0.86; *P* = .003) OS (overall population): 23.4 vs. 16.8 mo (HR 0.64; 95% CI, 0.49 to 0.84; *P* = .001)	HER2^b^ by IHC 1+ or 2+/ISH-
			HER2 + mGC or mGEJ adenoca, ≥3L (after 2 prior regimens including trastuzumab, a fluoropyrimidine and a platinum based CHT)	DESTINY-Gastric01 (II): randomized vs. TPC	ORR 51.3% vs. 14.3% (*P* < .0001)^f^	OS 12.5 vs. 8.9 mo (HR 0.60; 95% CI, 0.42-0.86)¤ PFS 5.6 vs. 3.5 months (HR 0.47; 95% CI, 0.31-0.71)¤ ORR 42.0% vs 12.5% (*P* = .0001)¤ DOR 12.5 vs. 3.9 mo¤ DCR 85.7% vs 62.5% (*P* = .0005)^f^	HER2^c^ by IHC 3+ or 2+/ISH+
			HER2-mutant unresectable/mNSCLC, ≥2L	DESTINY-Lung02 (II): randomized, non-comparative. Cohort 1: T-DXd 5.4 mg/kg q3w Cohort 2: T-DXd 6.4 mg/kg q3w	ORR (T-DXd 5.4 mg/kg): 53.8% ORR (T-DXd 6.4 mg/kg): 42.9%	DOR (T-DXd 5.4 mg/kg): NE (4.2-NE) DOR (T-DXd 6.4 mg/kg): 5.9 (2.8-NE) PFS; OS	HER2 activating mutation by NGS
FRα	Mirvetuximab soravtansine-gynx (MIRV)	Payload: DM4, microtubule inhibitor Linker: cleavable DAR: 3.5	FRα+ PROC, fallopian tube, primary peritoneal cancer progressing to 1-3 prior lines	SORAYA (II): single arm, not randomized	ORR 32.4% (95% CI, 23.6-42.2; *P* < .0001)	DOR: 6.9 mo (95% CI, 5.6-9.7)	FRα+ by IHC
				MIRASOL (III): randomized vs. TPC	PFS 5.62 vs. 3.98 mo (HR 0.65 95% CI, 0.52-0.81; *P* < .0001)	ORR 42.3% vs. 15.9% (p < 0.0001) OS 16.46 vs. 12.75 mo (HR 0.67 95% CI, 0.50-0.88; *P* = .0046)	
Trop-2	Sacituzumab govitecan (SG)	Payload: SN-38 (active metabolite of irinotecan), campothecin, TOPO1 inhibitor Linker: cleavable DAR: 8	mTNBC, after ≥2 lines of systemic therapies (at least 1 for mTNBC)	ASCENT (III): randomized vs. TPC	PFS 5.6 vs 1.7 mo (HR: 0.39; *P* < .0001)^g^	OS 12.1 vs 6.7 mo (HR: 0.48, *P* < .0001)^g^	None
			HR+/HER2- mBC, after ET and at least 2 additional CHT for mBC	TROPiCS-02 (III): randomized vs. TPC	PFS 5.5 vs. 4 mo (HR, 0.66; 95% CI, 0.53-0.83; *P* = .0003)	OS 14.4 vs 11.2 mo (HR, 0.79; *P* = .020)	None
			mUC, after platinum based-CHT and ICI	TROPHY U-01 (II): multicohort, open label, not randomized	ORR 28% (95% CI, 20.2-37.6)^h^	DOR 6.1 mo (95% CI, 4.7-9.7)^h^ PFS 5.4 mo (95% CI, 3.5-6.9)^h^ CBR 38% (95% CI, 29.1-47.7)^h^ OS 10.9 mo (95% CI, 8.9-13.8)^h^	None
TF	Tisotumab vedotin-tftv (TV)	Payload: MMAE, auristatin, microtubule inhibitor Linker: cleavable DAR: 4	Recurrent/mCC, after no more than 2L	InnovaTV 204 (II): single arm, not randomized	ORR 24% (95% CI, 16-33)	DOR 8.3 mo (95% CI, 4.2-NR) PFS 4.2 (95% CI, 3.0-4.4) OS 12.1 mo (95% CI, 9.6-13.9)	None
Nectin-4	Enfortumab vedotin (EV)	Payload: MMAE, auristatin, microtubule inhibitor Linker: cleavable DAR: 4	la/mUC, after platinum based-CHT and ICI	EV-301 (III): randomized vs. TPC	OS 12.91 vs 8.94 mo (HR 0.704, 95% CI, 0.581-0.852; *P* = .00015)^i^	PFS 5.55 vs. 3.71 mo (HR 0.632, 95% CI, 0.525-0.762; *P* < .00001)^i^ ORR 40.6% vs. 17.9% (*P* < .001) DCR 71.9% vs. 53.4% (*P* < .001) DOR 7.39 vs. 8.11 mo	None
			la/mUC, 1L in combination with pembrolizumab, for pts ineligible for cisplatin containing CHT	EV-103/KEYNOTE-869 (Ib/II): Cohort A: EV single arm, not randomized Cohort K: randomized EV + P vs. EV	Cohort K: ORR 64.5% vs.45.2%	Cohort A: ORR 73.3% (95% CI, 58.1-85.4) PFS 12.7 mo OS 26.1 mo^h^ Cohort K: DOR NR vs.13.2 mo	None

^a^Median value reported for DOR, OS, PFS.

^b^According to the ASCO/CAP HER2 testing in breast cancer guideline (2018).

^c^According to HER2 Testing and Clinical Decision Making in Gastroesophageal Adenocarcinoma: Guideline from the CAP-ASCP-ASCO (2016).

^d^Last update: 2021 ESMO Congress 2021.

^e^Updated results in 2023.

^f^Last update: 2022 ASCO Gastrointestinal Cancers Symposium.

^g^Last update: 2022 ASCO Annual Meeting I.

^h^Last update: 2023 ASCO Genitourinary Cancers Symposium.

^i^Last update: 2022 ASCO Annual Meeting I.

Abbreviations: FDA: Food and Drug Administration; Ag: antigen; ASCO: American Society of Clinical Oncology; CAP: College of American Pathologists; ASCP: American Society for Clinical Pathology; ESMO: European Society for Medical Oncology; HER2: human epidermal growth factor receptor 2; FRα: folate receptor alpha; Trop-2: trophoblast cell surface antigen-2; TF: tissue factor; T-DM1: ado-trastuzumab-emtansine; T-DXd: fam-trastuzumab deruxtecan-nxki; MIRV: mirvetuxumab soravtansine; MMAE: monomethyl auristatin E; TOPO1: topoisomerase 1; DAR: drug-to-antibody ratio; eBC: early breast cancer; mBC: metastatic breast cancer; mGC: metastatic gastric cancer; mGEJ adenoca: metastatic gastro-esophageal junction adenocarcinoma; mNSCLC: metastatic non-small cell lung cancer; PROC: platinum-resistant epithelial ovarian cancer; la/mUC: locally advanced/metastatic urothelial carcinoma; P: pembrolizumab; mCC: metastatic cervical cancer; L: therapy line; CHT: chemotherapy; HR: hormone receptor; TPC: physician’s choice chemotherapy; ET: endocrine therapy; ICI: immuno-checkpoint inhibitors; PFS: progression-free survival; OS: overall survival; ORR: objective response rate; DOR: duration of response; iDFS: invasive disease free survival; DRFI: distant recurrence-free interval; DCR: disease control rate; CBR: clinical benefit rate; HR: hazard ratio; CI: confidence interval; mo: months; NE: not estimable; IHC: immunohistochemistry; ISH: in situ hybridization; NGS: next-generation sequencing.

In this narrative review, we navigate the complex landscape of ADCs in solid cancers, evaluating examples of antigens’ targetability based on their expression and outline future perspectives of biomarkers of activity for ADCs.

### Human Epidermal Growth Factor Receptor 2

The receptor tyrosine-protein kinase erbB-2, or HER2, is a member of the epidermal growth factor receptor (EGFR) family. HER2 homodimerizes or heterodimerizes with other receptors of the same family (eg, human epidermal growth factor receptor 3, HER3), favoring the autophosphorylation of the intracellular tyrosine residues of the heterodimer and then triggering signaling pathways responsible for cellular proliferation and tumorigenesis.^16^ Owing to its oncogenic role, HER2 has been widely studied as a therapeutic target.^17^

#### HER2 in Breast Cancer

Around 15-20% of all breast cancers (BCs) overexpress HER2.^18^ According to the American Society of Clinical Oncology (ASCO)/College of American Pathologists (CAP) guidelines, BCs are defined as “HER2-positive” if HER2 expression by immunohistochemistry (IHC) is scored 3+ or 2+ with gene amplification detected by in situ hybridization (ISH). Tumors that do not meet these criteria are considered “HER2-negative”.^19^ Given the results of the DESTINY-Breast04 trial,^20^ ASCO/CAP guidelines on HER2 testing in BC were recently updated recommending the addition of a footnote to HER2 testing reports to underline the need for recognizing tumors expressing low levels of HER2: although “HER2-low” (IHC scores 1+ or 2+ and negative ISH) and “HER2-ultra low” (IHC score 0 with incomplete and faint staining in less than 10% of tumor cells) BCs^20,21^ are still not classified as new categories, they should be distinguished from tumors completely lacking HER2 expression (ie, no cell membrane staining) due to therapeutical implications in terms of eligibility for T-DXd.^22,23^ Considering the biological and clinical relevance of HER2 as a driver in BC, several HER2 targeting agents are currently approved for the treatment of HER2-positive BC, including ADCs.

##### Ado-trastuzumab-emtansine

T-DM1 has been the first FDA-approved ADC for solid tumors and consists of the anti-HER2 mAb trastuzumab conjugated via a stable linker to DM1, a microtubule inhibitor.^2^ Based on the results of the EMILIA and TH3RESA trials, T-DM1 became the standard of care for the treatment of HER2-positive metastatic breast cancer (mBC) in second line and beyond.^24,25^ Lately, T-DM1 has been established as standard treatment for patients with HER2-positive BC who do not achieve pathologic complete response (pCR) after neoadjuvant therapy (KATHERINE trial).^26^

Although T-DM1 has been mainly studied in HER2-positive BC, negative data regarding its activity in “HER2-low” BC are available. Burris et al evaluated T-DM1 in a phase II trial enrolling patients with HER2-positive mBC progressing after prior treatment with an anti-HER2 agent and chemotherapy. A retrospective reassessment of HER2 expression was done on archival primary tumors, finding a group of “HER2-negative” patients in which T-DM1 showed very modest activity (overall response rate, ORR, 4.8% and median progression-free survival, mPFS, 2.6 months) compared to patients with confirmed HER2-positive tumors (ORR 33.8% and mPFS 8.2 months). Otherwise, higher levels of HER2 expression evaluated by quantitative real time-polymerase chain reaction (qRT-PCR) were associated with better responses.^27^

In the KRISTINE trial, neoadjuvant T-DM1 plus pertuzumab did not improve pCR rates nor event-free survival (EFS) rates when compared to chemotherapy plus dual-HER2 blockade,^28^ although higher HER2 levels were associated to higher pCR rates, irrespective of treatment arm. In the T-DM1 plus pertuzumab arm, 15 patients had an EFS event during the neoadjuvant period, and all these tumors were found with lower and heterogeneous HER2 expression.^29,30^ These results suggest that an IHC score 3+ may select T-DM1-responders better than the traditional HER2 positivity definition, optimized for trastuzumab, limiting T-DM1 utility in HER2-low tumors.^31^ In fact, HER2 oncogene addiction seems still very relevant to result in clinical benefit for T-DM1, suggesting that the activity is related to the presence of the HER2 antigen on the cells, and partly related to the inhibition of HER2-dependent cancerous signaling.^32^

##### Fam-trastuzumab deruxtecan-nxki

T-DXd includes a cleavable linker, which connects trastuzumab to deruxtecan, a topoisomerase I inhibitor, with a drug-to-antibody ratio of 8:1, that is considerably higher than that of T-DM1 (3.5:1).^2^ T-DXd showed clinical benefit in patients with HER2-positive and HER2-low BC.^20,33^ In the DESTINY-Breast01,^34^ -02,^35^ and -03^33^ trials, T-DXd demonstrated efficacy in patients with pretreated HER2-positive mBC, gaining the FDA and the European Medicines Agency approval in this setting,^36^ becoming the new second-line standard of care.^37,38^ Relevantly, DESTINY-Breast02 is the first study demonstrating efficacy of an ADC (T-DXd) in patients previously treated with another ADC (T-DM1), suggesting that T-DXd could overcome resistance to T-DM1.^35^ Additional benefit was showed in HER2-low, pretreated mBCs,^39^ as confirmed by the phase III DESTINY-Breast04 trial, in which T-DXd outperformed physician’s choice chemotherapy in terms of mPFS and OS.^20^ Likewise, 2 other anti-HER2 ADCs, namely, trastuzumab duocarmazine^40^ and disitamab vedotin,^41,42^ demonstrated activity in HER2-low mBC, albeit in earlier phases of drug development.

T-DXd seems to be effective also in patients with pretreated “HER2 ultra-low” mBC, according to the preliminary results of the DAISY trial (Cohort 3, HER2-nul: IHC score 0), reporting a best overall response of 30.6%.^43^ Of note, differently from T-DM1’s payload, deruxtecan can cross cell membranes, so that T-DXd includes the by-stander effect among its mechanisms of action.^44^

##### HER2 as a Spectrum

Given the expression of HER2 as a spectrum rather than a binary variable, it is important to capture and report such a diversity in clinical trials, to grasp the multifaceted role of HER2 as a biomarker and overcome pitfalls related to its assessment. All BCs can express HER2 to some extent and subsets of cancer cells may express HER2 regardless the main tumor phenotype^45^; however, currently approved IHC assays were not designed for the assessment of such subtle distinctions across low levels of expression.^46,47^ IHC alone seems not reliable to differentiate HER2-low versus HER2-null samples: Fernandez et al found only a 26% concordance among 18 board-certified pathologists who were asked to differentiate HER2 IHC score 0 from 1+.^48^ At the same time, ISH test can identify *ERBB2* gene amplification, but it does not commonly portend HER2-low expression, and its use is only relevant for defining HER2 positivity as a legacy of the traditional dichotomous approach.^49^ Therefore, emerging quantitative assays, like measuring *ERBB2* messenger RNA (mRNA) may help at refining HER2-scoring, supporting more traditional approaches of HER2 status determination.^47,50^ A transcriptomic analysis found higher *ERBB2* mRNA levels in HER2-enriched BC subtypes identified by the PAM50 assay,^51^ and higher *ERBB2* mRNA levels have been associated with better response rates to HER2-targeted therapies in different studies over time.^27,52-56^ Xu et al confirmed that IHC ranges seem too limited to allow a correct HER2 evaluation when it comes to lower levels of expression. Still, a specific *ERBB2* mRNA threshold for HER2-low tumors identification is lacking. Xu’s study also found IHC and mRNA to be inconsistent in evaluating HER2 expression, as tumors with higher IHC scores did not show significantly greater *ERBB2* mRNA levels than those with lower IHC scores.^57^ Considering these discrepancies, further studies are necessary to validate *ERBB2* mRNA levels as surrogate of membrane antigen expression and ADCs efficacy.

##### Spatial and Temporal Heterogeneity

Relevant obstacles in HER2 interpretation are function of the intratumor heterogeneity, defined as the presence of neoplastic cell populations with different HER2-score within the same tumor specimen, and intertumor heterogeneity, in which HER2-status varies among different metastatic sites.^16^ Besides heterogeneity, HER2 status is also dynamic, as demonstrated by the loss or gain of HER2 expression in both early and metastatic setting,^58-60^ which may reflect tumor heterogeneity (ie, HER2 status is different between primary and metastatic tumor or between 2 different metastatic sites) but also the result of acquired resistance to previous treatments. BC can lose HER2-expression as a result of the selective pressure elicited by HER2-directed therapies^61^ or, in case of HR-positive/HER2-negative mBC, BC cells can upregulate HER2 expression as a mechanism of resistance to endocrine agents, converting into HER2-positive tumors.^62^ Therefore, rebiopsy and retest for pathology biomarkers on progressing lesions, including HER2, are crucial for some patients showing treatment resistance or a different disease trajectory, to capture potential heterogeneity and phenotype switch.^63^

##### Molecular Imaging

Pathology may be still too partial in the definition of the overall tumor heterogeneity, as the information retrieved by tissue biopsies is piecemeal, and not able to be comprehensive. Therefore, molecular imaging has the potential to overcome such limitations, with the use of marked HER2 antibodies that can in vivo track HER2 expression. In the ZEPHIR trial, higher HER2 expression levels detected by HER2-positron emission tomography (PET)/computed tomography (CT) with ^89^Zr-trastuzumab (zirconium-positron-emitting radionuclide trastuzumab) were associated with a longer median time-to-treatment-failure in patients with HER2-positive mBCs treated with T-DM1, suggesting that the HER2-PET/TC could be a valid tool to study inter-lesions heterogeneity and complement standard pathological HER2 assessment (ie, IHC and ISH) to select T-DM1 responders.^64^ Pertuzumab, labeled with ^89^Zr, has also been used to trace HER2 expression in patients, representing a sensitive way of HER2 detection without any competition for the trastuzumab-binding-epitope (domain IV), as it binds to a different one (domain II).^65^ This may be important, when patients are under treatment with trastuzumab, to ensure a reliable portray of the HER2 status, and minimize the need for wash-out. ^89^Zr-pertuzumab could recognize patients with HER2-positive breast cancer metastases whose primary tumors were HER2-negative, resulting in potential therapeutic effects.^66,67^ Since HER2-PET/TC assesses HER2 distribution among all metastatic sites simultaneously, it allows to overcome some limitations related to biopsies that, besides being more invasive procedures, permit to evaluate just one metastatic sample at a time.

##### Circulating Tumor Cells and Circulating Tumor DNA

**Figure 3.**
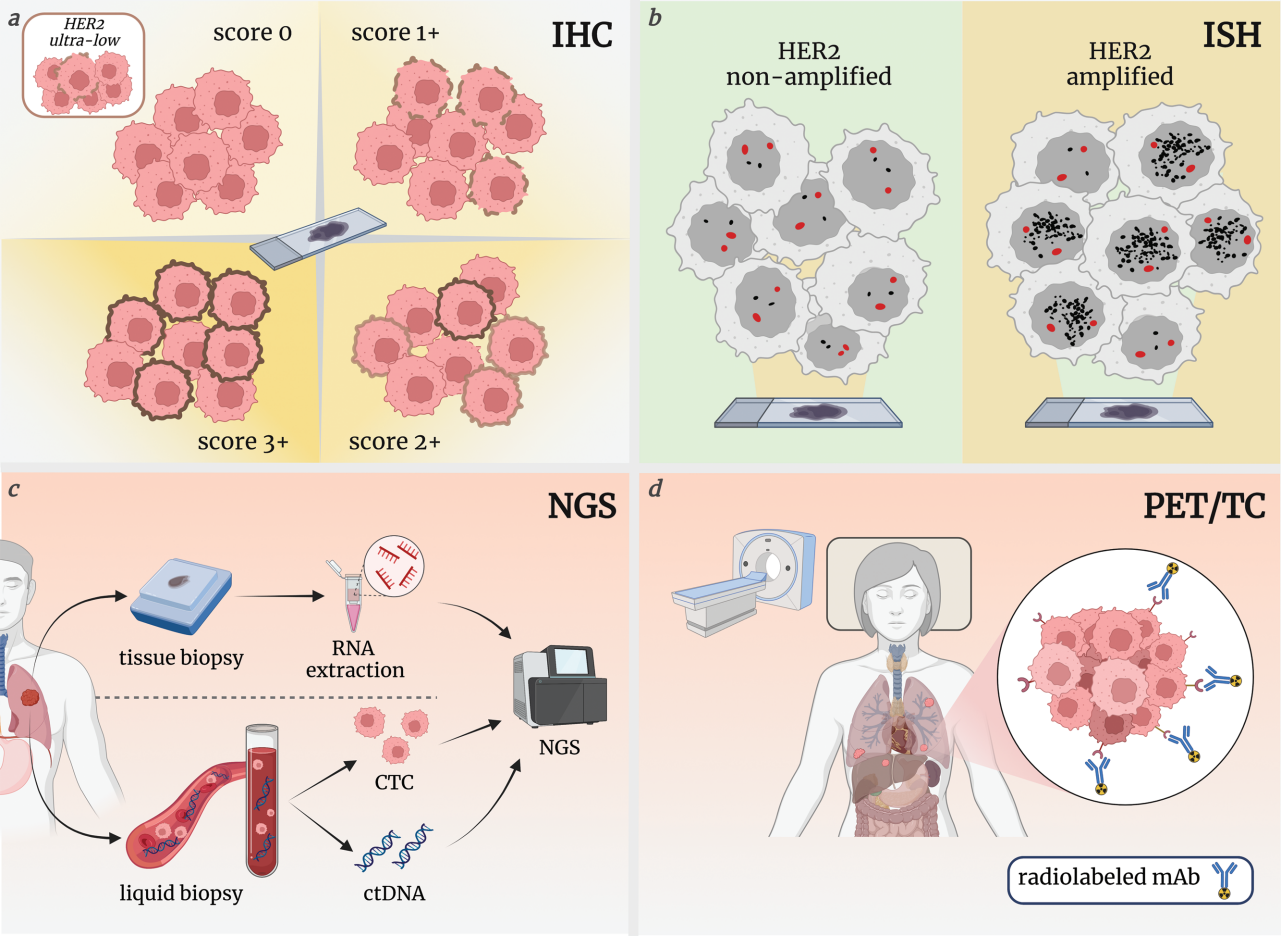
HER2 expression assessment methods. (**A**) IHC evaluates protein expression on tumor cell membrane. According to ASCO/CAP guidelines for HER2 testing in breast cancer, IHC score 0 consists of no staining or membrane staining that is incomplete and is faint/barely perceptible and in ≤10% of tumor cells, the latter is already known as “HER2-ultra low” (top-left); IHC score 1+ describes an incomplete membrane staining that is faint/barely perceptible and in >10% of tumor cells (top-right); IHC score 2+ (equivocal) is a weak to moderate complete membrane staining observed in >10% of tumor cells (bottom-right); IHC score 3+ is defined by circumferential membrane staining that is complete, intense and in >10% of tumor cells (bottom-left). (**B**) ISH identifies ERBB2 gene amplification using a silver precipitate (SISH) or a fluorescent chromogen (FISH), it is ordered in case of IHC score 2+ “equivocal.” VENTANA HER2 Dual-ISH (DISH) labels the ERBB2 gene (black dots) and chromosome 17 (red dots) with chromogenic probes detected by SISH and red-ISH, respectively, using light microscopy. HER2 status is determined by the ratio between ERBB2 gene over chromosome 17 signals. Source: Biomarker Testing of Specimens from Patients with Carcinoma of the Breast, v1.5.0.1, available at: www.cap.org/cancerprotocols. (**C**) NGS is a high-throughput technique for gene expression analysis and quantification of RNA levels. RNA is isolated from tissue samples (FFPE). ERBB2 mRNA levels could become complementary to IHC and refine HER2 assessment, especially for “HER2-low” and “HER2-ultra low” tumors. NGS can be also carried out on liquid biopsy samples, both on CTC and ctDNA, also representing a non-invasive alternative to monitor the plasticity of the target antigen expression throughout disease evolution. Other techniques to molecularly characterize CTC include FISH, PCR, CGH, and IF. PCR can be also performed for ctDNA testing. (**D**) HER2-PET/TC is a molecular imaging technique that evaluates HER2 expression on tumor cells using an anti-HER2 mAb (eg, trastuzumab or pertuzumab) labeled with radiometals (eg, Zirconium-89). It assesses HER2 expression across all the metastatic sites at the same time, representing a strategy to overcome limitations in HER2 evaluation related to tumor heterogeneity. Abbreviations: IHC: immunohistochemistry; HER2: human epidermal growth factor receptor 2; ISH: in situ hybridization; NGS: next-generation sequencing; mAb: monoclonal antibodies; ASCO: American Society of Clinical Oncology; CAP: College of American Pathologists; CTC: circulating tumor cells; ctDNA: circulating tumor DNA; FISH: fluorescence in situ hybridization; PCR: polymerase chain reaction; CGH: comparative genomic hybridization; IF: immunofluorescence; PET-TC: positron emission tomography-computed tomography; FFPE: formalin-fixed paraffin embedded. Created with BioRender.com (2023).

Another non-invasive alternative for monitoring HER2 dynamic could be liquid biopsy (Fig. 3). The CirCe T-DM1 trial showed a very limited efficacy of T-DM1 in patients with pretreated HER2-negative mBC, and HER2 amplification found on CTC: a strong limitation of this approach is the need for detecting a sufficient number of CTC to test biomarkers of interest.^68^ On the other side, biomarker analysis on ctDNA is emerging as a tool to monitor tumor response and select patients for ADCs.^69^

##### HER2: Binary Versus Quantitative Biomarker

Presently, it is unclear what and if a minimum expression of HER2 is important to portend therapeutic benefits with T-DXd.^44^ HER2 expression is progressively moving from being a “binary biomarker” with dualistic implications to be a “quantitative biomarker” across a spectrum, that deserves a continuous scoring system, highlighting the need to find trustworthy methods for the precise quantification of its expression to better guide treatment choices (Fig. 3).

#### HER2 in Non-Small Cell Lung Cancer

HER2 molecular alterations can be also found in NSCLC. HER2 amplification is present in 1%-3% of cases, while HER2 hyperexpression in 2%-38%. HER2 mutations are reported in 1%-4% of NSCLCs, the most common involving the intracellular domain and being in-frame insertion mutations in exon 20 (eg, A775_G776insYVMA).^70,71^ Both pan-HER tyrosine kinase inhibitors (TKIs) and selective TKIs have limited use in HER2-mutant NSCLCs, due to the poor efficacy showed by the former and the major toxicity brought by the latter.^72-78^ Similarly, anti-HER2 mAb have limited efficacy when used in monotherapy, with some activity shown only in association with chemotherapy.^79-81^

Interest in targeting HER2 alterations in NSCLC made a comeback thanks to ADCs.^82^ T-DM1 was evaluated in HER2-overexpressing NSCLC with unsatisfying results, especially in patients with HER2 IHC 2+,^83^ while promising results were seen in patients with HER2-mutant NSCLC (ORR: 44%), irrespectively of the mutation subtypes and HER2-protein expression levels.^84^

In the DESTINY-Lung01 trial, T-DXd was investigated in recurrent/refractory HER2 IHC 2/3+ overexpressing (cohort 1) and HER2 mutant (cohort 2) NSCLC. Cohort 2 reported better outcomes (ORR: 55%; mPFS: 8.2 months; mOS: 17.8 months) as compared to cohort 1 (ORR: 24.5%; mOS: 11.3 months).^85,86,87^ Responses were observed across all mutation subtypes, including intracellular and kinase domains, and, notably, in most of those tumors with no detectable HER2-expression or *ERBB2* gene amplification. Therefore, in NSCLC, it seems that mutations are the HER2-aberrations associated with benefit from T-DXd, in contrast to breast and gastric cancers where HER2 expression levels represent the main biomarker for patient selection.^34,88^ Preclinical studies found that, in NSCLC, *ERBB2* mutations and amplifications do not generally overlap, possibly representing 2 distinct therapeutic targets,^89,90^ although molecular imaging showed radiolabeled trastuzumab accumulating in both *ERBB2* amplified and mutant NSCLC. As such, the different T-DXd activity shown in the 2 cohorts of the DESTINY-Lung01 still has to be elucidated: a possible explanation resides in HER2-mutant cells having high-rates of receptor ubiquitination and internalization, which predicted a more effective endocytosis of HER2-ADCs complexes,^91^ favoring the intracellular delivery of the cytotoxic payload. On August 2022, FDA granted accelerated approval to T-DXd at the dose of 5.4 mg/kg q3w for HER2-mutant NSCLC,^92^ which showed less toxicity without jeopardizing efficacy when compared to the 6.4 mg/kg q3w dose in the DESTINY-Lung02.^93^

#### HER2 in Other Tumor Types

T-DXd efficacy has been consistently reported in HER2-positive metastatic gastric cancer both in the Asiatic (DESTINY-Gastric01 trial)^88^ and in the Western population (DESTINY-Gastric02 trial),^94^ with preliminary data of safety and tolerability with fluoropyrimidine combinations (DESTINY-Gastric03 trial).^95^ The results shown by T-DXd in BC, NSCLC, and gastric cancer promoted its investigation in other HER2-expressing tumors.

In a limited cohort of pretreated patients with multiple HER2-expressing tumors, T-DXd showed promising antitumor activity (ORR 40.9%, mPFS 11.1 months).^96^ The DESTINY-PanTumor02 trial (NCT04482309) is evaluating T-DXd in a larger pretreated population of multiple HER2-expressing advanced solid tumors: recently, a press release announced that the study met prespecified criteria for ORR and duration of response, and published results are awaited.

##### HER2, a Controversial Biomarker Across Histologies

Difficulties in defining which HER2 alteration can best predict ADCs activity and the variable outcomes shown by T-DXd (and ADCs in general) when evaluated among different histotypes make HER2 a quite controversial biomarker. The explanation for such variability could be found in the peculiar biology of each single tumor (eg, tumors rich in fibrosis that limits the bystander effect, presence of various concomitant oncogenic drivers, different sensitivity to the cytotoxic payload released). These aspects should be further investigated to reshape HER2 role as a predictive biomarker and better select patients for treatments.

### Folate Receptor Alpha

Folate Receptor Alpha (FRα) is a cell surface glycoprotein responsible for folate entrance into the intracellular environment and limitedly expressed in normal tissues. As folate is necessary for DNA synthesis and cellular proliferation, FRα is upregulated when cellular metabolic demand increases, that is typical of cancer cells.^97,98^ FRα is overexpressed in different cancers, especially high-grade serous ovarian cancer (OC), and its expression remains constant without being influenced by systemic therapies, thus representing an ideal therapeutic target.^99^

#### FRα in Ovarian Cancer

MIRV is an ADC conjugating an anti-FRα antibody to the tubulin disruptor maytansinoid DM4, via a cleavable linker.

FRα expression can be tested by IHC using FOLR1-2.1, a murine mAb. After proving activity in patient with FRα-positive (IHC 2+ in ≥25% of tumor cells) platinum-resistant OC (ORR 26%),^100^ MIRV outcomes were evaluated according to FRα expression, demonstrating that high-FRα levels (IHC 2+ in ≥75% of tumor cells) were associated to better ORR.^101^

MIRV efficacy in patients with FRα-high platinum resistant OC was specifically evaluated in the single arm phase I SORAYA trial; the study met its primary endpoint achieving an ORR of 32.4%,^102^ leading the FDA approval of MIRV for patients with pretreated FRα-positive, platinum resistant OC, fallopian tube, or primary peritoneal cancer.^103^ Importantly, in the FORWARD I, a phase III trial enrolling patients with FRα-positive platinum resistant OC, MIRV did not improve PFS compared with single agent chemotherapy in the intention to treat population but proved better activity only in patients with high-FRα expression.^104^ Hence, a comparison between MIRV efficacy and investigator’s choice chemotherapy in tumors expressing high-FRα levels (FRα by IHC ≥ 75%) was specifically addressed in the MIRASOL phase III trial (NCT04209855), in which MIRV guaranteed a statistically significant improvement in PFS and OS.^105^ MIRV is the first biomarker-driven ADC to become a standard of care for OC: results from the MIRASOL trial suggest that FRα expression cutoffs improve patients’ selection for MIRV, despite a cleavable linker and a demonstrated by-stander effect of DM4 metabolites.^106,107^

### Trophoblast Cell Surface Antigen-2

Trophoblast cell surface antigen-2 (Trop-2) is a transmembrane glycoprotein involved in tumor growth stimulation, encoded by the *TACSTD2* gene.^108^ It is relatively poorly represented on normal tissues but highly expressed by a wide variety of solid tumors. Data suggest that its overexpression represents a negative prognostic biomarker in some tumors.^109^ Being largely expressed in tumors, Trop-2 has been investigated as a therapeutic target.

#### Trop-2 in BC

Trop-2 is expressed in more than 90% of BC, especially triple-negative BC (TNBC).^110^ The ADC sacituzumab ­govitecan (SG) conjugates an anti-Trop-2 mAb with a topoisomerase I inhibitor, SN-38, an active metabolite of irinotecan. SG proved efficacy in both metastatic TNBC (mTNBC)^111,112^ and HR-positive/HER2-negative mBC^113^, unselected for Trop-2 expression.^112^Findings from post hoc analyses evaluating SG efficacy according to Trop-2 expression in mBC weaken the role of the employed Trop-2 cutoffs as predictive of response to SG (Table 3).^114,115^ In fact, FDA approved SG for both mTNBC and HR-positive/HER2-negative mBC regardless Trop-2 expression.^116^ In the phase I TROPION-PanTumor01 study, datopotamab deruxtecan (Dato-DXd), a novel ADC conjugating an anti-Trop-2 antibody with deruxtecan, showed activity in pretreated patients with mTNBC not selected by Trop-2 expression.^117^

**Table 3.** Selected biomarker analyses evaluating SG efficacy according to Trop-2 expression.

Drug	Target Ag	Assay	Post-hoc analyses (year)	Trial (design)	Setting	Trop-2 expression categories (H-score)	Efficacy outcomes according to target expression^a^ (SG vs. TPC)	Notes
SG	Trop-2	IHC (H-score^b^)	Bardia et al	ASCENT (SG randomized vs. TPC)	mTNBC	Trop-2 low 0-100	PFS 2.7 vs. 1.6 mo OS 9.3 vs. 7.6 mo	Outcomes were numerically higher with SG vs. TPC in pts with high and medium Trop-2 expression. The restricted number of pts with low Trop-2 expressing tumors precludes a definitive conclusion on whether SG efficacy is limited by low Trop-2 expression.
						Trop-2 medium 100-200	PFS 5.6 vs. 2.2 mo OS 14.9 vs. 6.9 mo	
						Trop-2 high 200-300	PFS 6.9 vs. 2.5 mo OS 14.2 vs. 6.9 mo	
			Rugo H. et al (2023)	TROPiCS-02 (SG randomized vs. TPC)	HR+/HER2- mBC	H-score <100	H-score < 100 overall: PFS 5.3 vs. 4.0 mo (HR 0.77) OS 14.6 vs. 11.3 mo (HR 0.75) H-score ≤ 10 subgroup: PFS 5.5 vs 4.3 mo (HR 0.89) OS 17.6 vs. 12.3 mo (HR 0.61) H-score > 10-<100 subgroup: PFS 5.1 vs. 3.5 mo (HR 0.67) OS 13.7 vs. 11.0 mo (HR 0.81)	SG outperformed TPC regardless of Trop-2 expression, although using different Trop-2 expression cutoffs than that of Bardia et al. No specific level of Trop-2 expression at which SG showed an improved effect has been identified in this post hoc analysis.
						H-score ≥100	PFS 6.4 vs. 4.1 mo (HR 0.60) OS 14.4 vs. 11.2 mo (HR 0.83)	

^a^Median value reported for OS and PFS.

^b^The H-score is used to categorize Trop-2 expression on tumor cell membranes, once evaluated by IHC. The score is based on the staining intensity and on the percentage of tumor cells stained at that intensity.

Abbreviations: Ag: antigen; H-score: histochemical score; SG: sacituzumab govitecan; TPC: treatment of physician’s choice; Trop-2: trophoblast cell-surface antigen 2; IHC: immunohistochemistry; mTNBC: metastatic triple negative breast cancer; HR: hormone receptor, HER2: human epidermal growth factor receptor 2; mBC: metastatic breast cancer; PFS: progression-free survival; OS: overall survival; mo: months; pts: patients.

#### Trop-2 Across Histotypes

In the basket IMMU-132-01 phase I trial, SG showed signs of activity across histologies,^118^ encouraging SG testing in different tumors other than BC. The phase II TROPHY-U-01 study confirmed SG clinical activity in patients with pretreated metastatic urothelial cancer, leading to the FDA-accelerated approval of SG in this population (Table 2).^119^ Of note, in all the settings in which SG is approved, Trop-2 expression is not required. However, a TNBC cell line expressing low Trop-2 levels (MDA-MB-231) showed scarce sensitivity to SG, in contrast, SN-38 administered as a free-cytotoxic agent achieved relevant therapeutic effects in the same cell line: once proved the sensitivity to the cytotoxic payload, a possible culprit for the impairment of SG efficacy in the MDA-MB-231 cell line may be identified in low levels of Trop-2 expression.^120^ In this regard, the TROPiCS-03 phase II trial is testing SG in tumors with elevated Trop-2 expression to further elucidate Trop-2 role in predicting response to SG (NCT03964727).

### Nectin-4

Nectin-4 is a transmembrane protein involved in cellular adhesion. It is aberrantly expressed in cancer cells, especially urothelial cancer. Enfortumab vedotin (EV) is an approved anti nectin-4 antibody and a microtubule inhibitor conjugated.^121^

#### Nectin-4 in Urothelial Cancer

The EV-101 phase I trial evaluating EV in solid tumors initially required nectin-4 expression by IHC for the inclusion; later on, the protocol was amended to remove this requirement, as nectin-4 was found ubiquitously expressed in urothelial cancers screened.^122^ Accordingly, in the pivotal phase II (EV-201^123^) and in the confirmatory phase III (EV-301^124^) trials, nectin-4 expression was evaluated just for exploratory purposes. In fact, EV has been approved by FDA for the treatment of advanced/metastatic urothelial cancer without any prior assessment for nectin-4 expression^125,126^ (Table 2). However, a decrease of nectin-4 membranous expression in metastatic urothelial cancer samples compared to matched primary samples has been recently described, possibly representing a mechanism of resistance to EV, predicting unfavorable outcomes, and leaving an open question regarding the inclusion of nectin-4 status as a biomarker to improve patients’ selection for EV.^121^

### Receptor Tyrosine-Protein Kinase erbB-3 (HER3)

HER3 is a membrane enzyme encoded by the *ERBB3* gene.^127^ Its most studied ligands are Neuregulin-1 and Neuregulin-2, which can bind to the extracellular domain of the receptor.^127,128^ Because HER3 lacks the kinase activating domain, its activity is carried out by heterodimerization with other HER-family receptors.^129^ HER3 overexpression is reported in various tumors, including breast and lung cancer, and correlates with poor prognosis.^130-132^ Patritumab deruxtecan (HER3-DXd) is the first-in-class HER3 targeting ADC, but other ADCs are under early development (eg, DR-1310).^133,134^

#### HER3 in NSCLC

HER3 is expressed in 82.7% of primary NSCLC.^135^ Recent evidence suggest that anti-EGFR TKIs increase HER3 expression in EGFR-mutant NSCLC, enhancing HER3-DXd activity.^136^ In the phase I trial NCT03260491, patients with EGFR-mutant NSCLC progressing to an anti-EGFR TKI received HER3-DXd achieving a mPFS of 8.2 months, while mOS was not reached; ORR was 39%, indicating an interesting anticancer activity of the compound. Pretreatment HER3 expression was assessed by IHC and found in all patients, with a median histochemical score (H-score) of 180. Responses to HER3-DXd were observed across a wide range of HER3 membrane expression levels, even if a trend to increased responses was seen for higher H-scores.^137^

#### HER3 in BC

HER3 has been associated with resistance to endocrine therapy in BC.^138^ HER3-DXd is being tested in BC, in both early and metastatic setting. Updated results of the phase I/II trial U31402-A-J101 confirmed the activity of HER3-DXd in HR-positive mBC and TNBC^139^; patients were enrolled in different dose expansion cohorts, according to HER3 expression by IHC: HER3 high and HER3 low HR-positive/HER2-negative mBC or HER3-high TNBC. HER3-high were defined as having ≥75% membrane positivity, while HER3-low tumors showed a membrane positivity between 25% and 75%.^139^ Preliminary results demonstrated no difference in terms of activity in patients with HR-positive mBC with either HER3-high or -low expression.^140^ Recently, HER3-DXd also showed early signs of activity in the phase II trial ICARUS-BREAST01 (NCT04965766), an ongoing study enrolling patients with pretreated HR-positive, HER2-negative/-low advanced BC, unselected for HER3 expression.^141^ Regarding early BC, results of the TOT-HER3 Part B trial showed that a single dose of HER3-DXd granted clinical response in HER2-negative early BC irrespective of *ERBB3* levels,^142^ in line with previous findings from Part A of the same trial, in which clinical response to HER3-DXd seemed not influenced by pre-treatment *ERBB3* mRNA levels.^143^

Overall, available data suggest that the activity of HER3-DXd does not directly correlate with HER3 expression but, deriving from phase I/II trials, these findings need validation in larger randomized clinical trials.

## Discussion and Conclusions

Target antigens can represent biomarkers of response to ADCs in some cases, and in some extent, but their clinical implementation has been so far very limited. Clinical trials with HER2-directed agents have initially stuck to the paradigm of antigen/antibody. However, the advent of agents capable to exert bystander activity have reshaped such paradigm (Table 2). Considering that, by definition, an antibody binds to cancer-antigens, albeit minimally expressed, and the bystander effect is complementary of the activity, the mechanisms of action of ADCs are yet to be fully deciphered. Presently, we are far from interpreting the mechanism of action of ADCs beyond the paradigm of “c*orpora non agunt nisi fixata*.” The challenge of this era is not to overlook antigens and broadly the biomarkers for ADCs, but to improve the capacity to identify patients who will derive the greatest benefit, based on novel biomarkers. This is a way to be paved with innovative approaches, including new diagnostics.

Obstacles found in predicting response to ADCs partly reflect the complex structure of these compounds, making necessary to search further beyond the target antigen and the by-stander effect but also consider other aspects such as the different sensitivity of each tumor histotype to the cytotoxic payload delivered or, else, the drug distribution that can be favored or not by specific histologic characteristics (eg, scarce/abundant fibrosis, rich/poor vascularization). In this rapidly evolving therapeutic scenario, designing biomarker-based clinical trials is becoming essential for treatment choice and prioritization, especially in those settings for which different agents proved efficacy and are approved. New techniques, including molecular imaging and gene expression, integrated with artificial intelligence for automated analyses will favor the achievement of accuracy and consistency for the evaluation of predictive biomarkers.^144^ The next generation of ADCs drug development must be precision drug development, and a shared effort between academia and pharmaceutical companies is needed to identify the subgroups of patients who can really benefit from the treatment with ADCs.

## Data Availability

No new data were generated or analyzed in support of this research.
